# Commencement

**Published:** 1881-04

**Authors:** 


### COMMENCEMENT.

The sixth annual commencement of the Dental College of the University of Michigan, was held in the hall of the University, on Wednesday, March 23rd, at 2 o'clock P. M.

The annual address was delivered by Dr. F. H. Rehwinkel, of Chillicothe, Ohio. A large and appreciative audience was present.

Upon the following named persons the degree of Doctor of Dental Surgery was conferred by the President of the University, Dr. Henry S. Frieze:

Wilbert George Bean..............................Detroit, Michigan.
Henry Franklin Billmeyer............................Brooklyn, Michigan.
Albert Victor Bills.................................Oakland, California.
Ephraim David Brower..................................Ackley, Iowa.
Joseph Burger..........................................Herzburg, Bavaria.
Solon Orville Burrington, M. D............Milwaukee, Wisconsin.
Charles Robert Calkins..............................Perry, New York.
George Henry Corey...................................Bristolville, Ohio.
Henry C. Corns..........................................Detroit, Michigan.
Lewis Craine....................................Erie City, Pennsylvania.
Hiram DePuy.................................Pittsburgh, Pennsylvania.
Alban Vaughan Elliott.........Washington, District of Columbia.
Almos Elias Emminger..........................Germantown, Ohio.
Fred. N. Emrick.......................................Germantown, Ohio.
Orion Jonathan Fay...............................Flat Rock, Michigan.
Stephen Humbert Gerow...............St. Johns, New Brunswick.
William Milo Hunt.......................................Columbus, Ohio.
Augustus Niel Johnson ..........................Newaygo, Michigan.
Edward Lincoln Kellogg...............................Atchison, Kansas.
Jennie Catherine Kollock..............................Chicago, Illinois.
John James Little...............................Keepville, Pennsylvania.
Charles Maclean.....................................Ann Arbor, Michigan.
Guy Hamilton Morgan..........West Bridgewater, Pennsylvania.
Denton E. Peterson...................................Waterloo, New York.
Charles Jay Siddall.........................................Oberlin, Ohio.
Charles Alfred B. Sipe.......................................Galion, Ohio.
John Silvus Tucker.........................................El Paso, Illinois.
Joseph William Wassall.................Mineral Point, Wisconsin.
Howard L. West...................................West Fairlee, Vermont.
B. Clark Williams...........................New Philadelphia, Ohio.

